# Supplementary material for: Single-cell-level digital twins for preterm birth prevention strategies
Source: bioRxiv. 2025 Oct 1:2025.09.29.679252. Preprint. [Version 1] doi: 10.1101/2025.09.29.679252 (PMC12621756; doi:10.1101/2025.09.29.679252)
Supplement: Supplement 1 — Figure S1: Detailed SIMCO workflow. Figure S2: Gating strategy. Figure S3: 96 well barcoding. Figure S4: All drug effect heatmaps. Figure S5: Comparison of SA, LPZ, and SALPZ drug effects. Figure S6: DMSO modeling. Table S1: Antibody panel. Table S2: CellOT performance in cross-validation across stimulation conditions. Table S3: Predictive performances for SIMCO-simulated data, Stelzer et al., and EGA. Table S4: Grid search parameters for Stabl/XGBoost models. [file media-1.pdf]

Single-cell-level digital twins for preterm birth prevention strategies

Supplementary data

Figure S1 Infographic depicting the SIMCO workflow in detail.
Figure S2 Unified gating strategy for mass cytometry of the perturbation dataset and the clinical dataset by Stelzer et al. 2021.
Figure S3 Design of the 96-well barcoding scheme.
Figure S4 Overview heatmaps of drug effects achieved by all drug candidates.
Figure S5 Synergistic, additive, and antagonistic effects of SA, lansoprazole, and SALPZ.
Figure S6 Modeling of time-dependent effects in signaling response across columns.

Table S1 Antibody panel of the perturbation atlas mass cytometry experiments.
Table S2 CellOT performance in cross-validation across stimulation conditions.
Table S3 Predictive performances for simulated data by SIMCO, original data by Stelzer et al. 2021, and EGA.
Table S4 List of hyperparameter grid search for Stabl/XGBoost models.

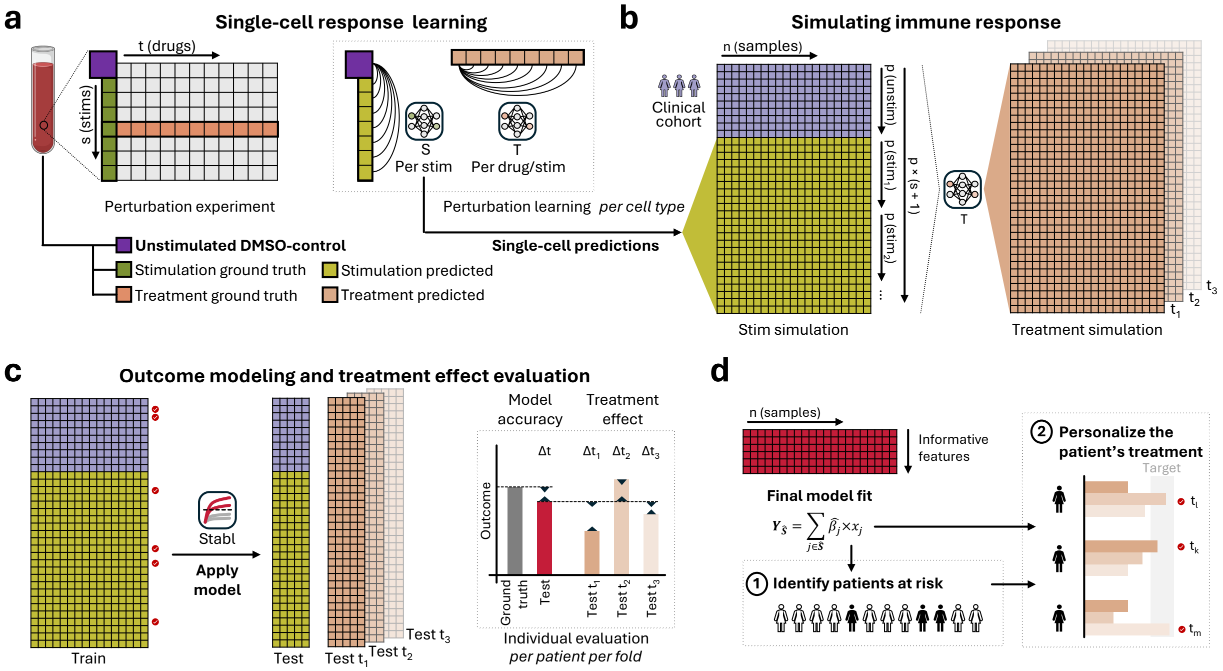

Figure S1 | Infographic depicting the SIMCO workflow in detail.

a. From a whole blood sample, perturbation experiments are performed to generate ground truth single-cell data (e.g., mass cytometry) over a matrix of s stimulations and t drug treatments.

b. Per cell type, CellOT models S_v and T_k are trained to learn perturbation responses to each stimulation v and to each treatment k (within a specific stimulation condition).

c. From an independent clinical cohort, n baseline samples (i.e., unstimulated and unexposed to treatments) are analyzed with mass cytometry, providing p features per sample. The resulting baseline dataset is expanded to include v stimulation scenarios through CellOT single-cell predictions, resulting in a simulated dataset containing (v +1) x p features. Using the stimulation-specific T_k CellOT models, a final dataset is built simulating single-cell treatment responses for each stimulation condition. The simulated treatment-response dataset contains (v +1) x p x t features.

d. The Stabl algorithm is then used to build a multivariable model predictive of a given clinical outcome (e.g., the time to labor onset). The expanded dataset (before simulated treatment) is split into a training and testing cohorts for cross-validation. Stabl selects clinical outcome-informative features and builds a multivariable model on the training cohort. The resulting Stabl model is then applied to the testing cohort and its treatment-simulated datasets t_k.

e. Model accuracy is evaluated as the difference between true and predicted outcome on the testing cohort. Treatment effects are evaluated per patient per cross-validation fold as the difference between the predicted outcome on the untreated data and the treatment-stimulated data.

f. The final Stabl model can be used to identify patients at risk for a given clinical outcome, and evaluation on the simulated-treatment responses can help personalize the patient's treatment.

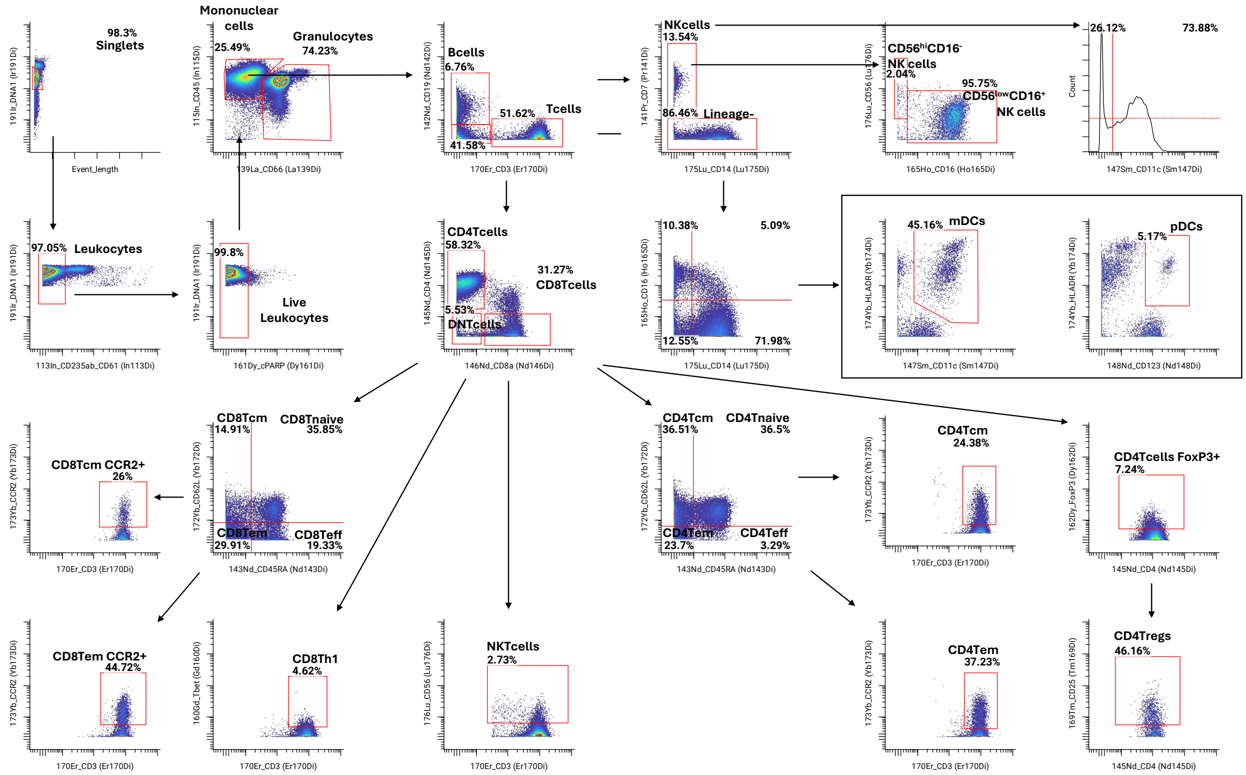

Figure S2 | Unified gating strategy for mass cytometry of the perturbation dataset and the clinical dataset by Stelzer et al. 2021. A total of 28 cell populations was manually gated and used for the analysis.

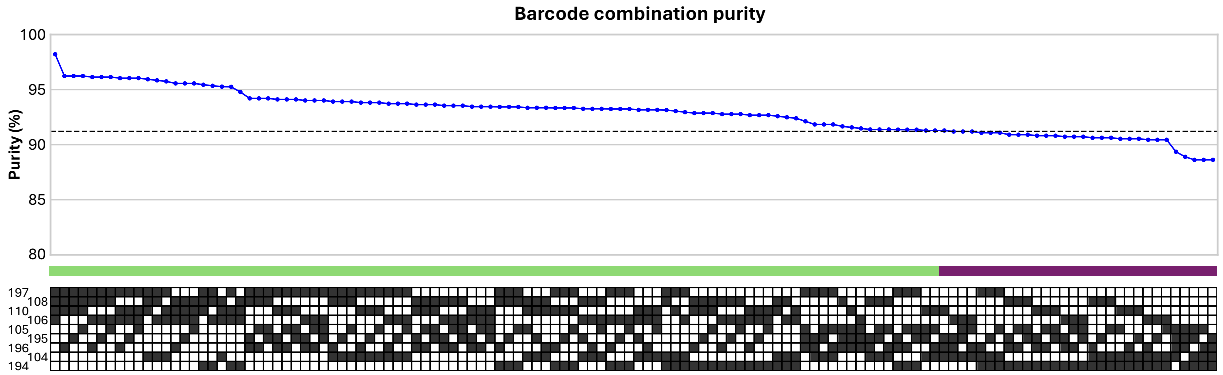

b

Isotope	Purity
Au197	99.9
Pd108	99.5
Pd110	99.4
Pd106	99.3
Pd105	98.8
Pt195	97.3
Pt196	97.3
Pd104	97.0
Pt194	96.5

c

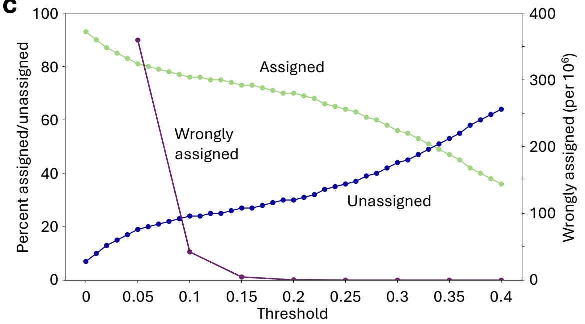

Figure S3 | Design of the 96-well barcoding scheme. Palladium (104, 105, 106, 108, 110), platinum (194, 195, 196) and gold (197) isotopes were used to construct a nine-choose-four barcoding system.

a. The theoretical purity of each of 126 possible barcoding labels calculated as product of the individual isotopes' purities. The 96 purest barcodes (green) were used, while the 30 most impure barcodes (purple) served as control. The threshold between used and unused barcodes was a purity of 91.28 %.

b. Table of isotope purities based on the vendor's specifications.

c. Varying the debarcoding threshold demonstrated how lower thresholds achieve higher assignment rates, while assigning more cells to unused ("wrong") barcodes (purple).

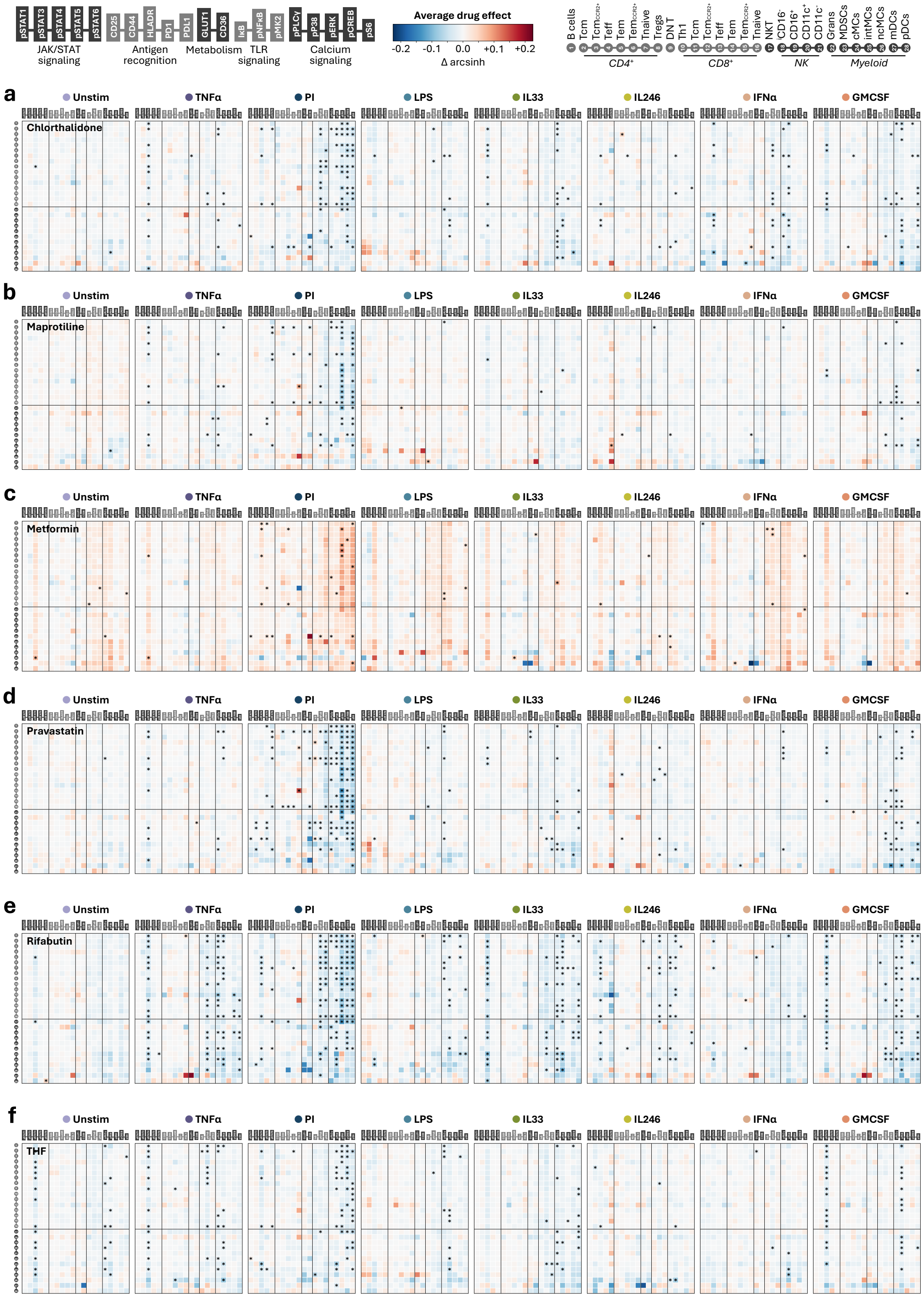

Figure S4 | Overview heatmaps of drug effects achieved by all drug candidates. Average drug effects across n = 11 pregnant individuals for chlorthalidone (**a**), maprotiline (**b**), metformin (**c**), pravastatin (**d**), rifabutin (**e**), and 5-methyl-tetrahydrofolate (THF, **f**). For SA, SALPZ, and lansoprazole see **Figure S5**.

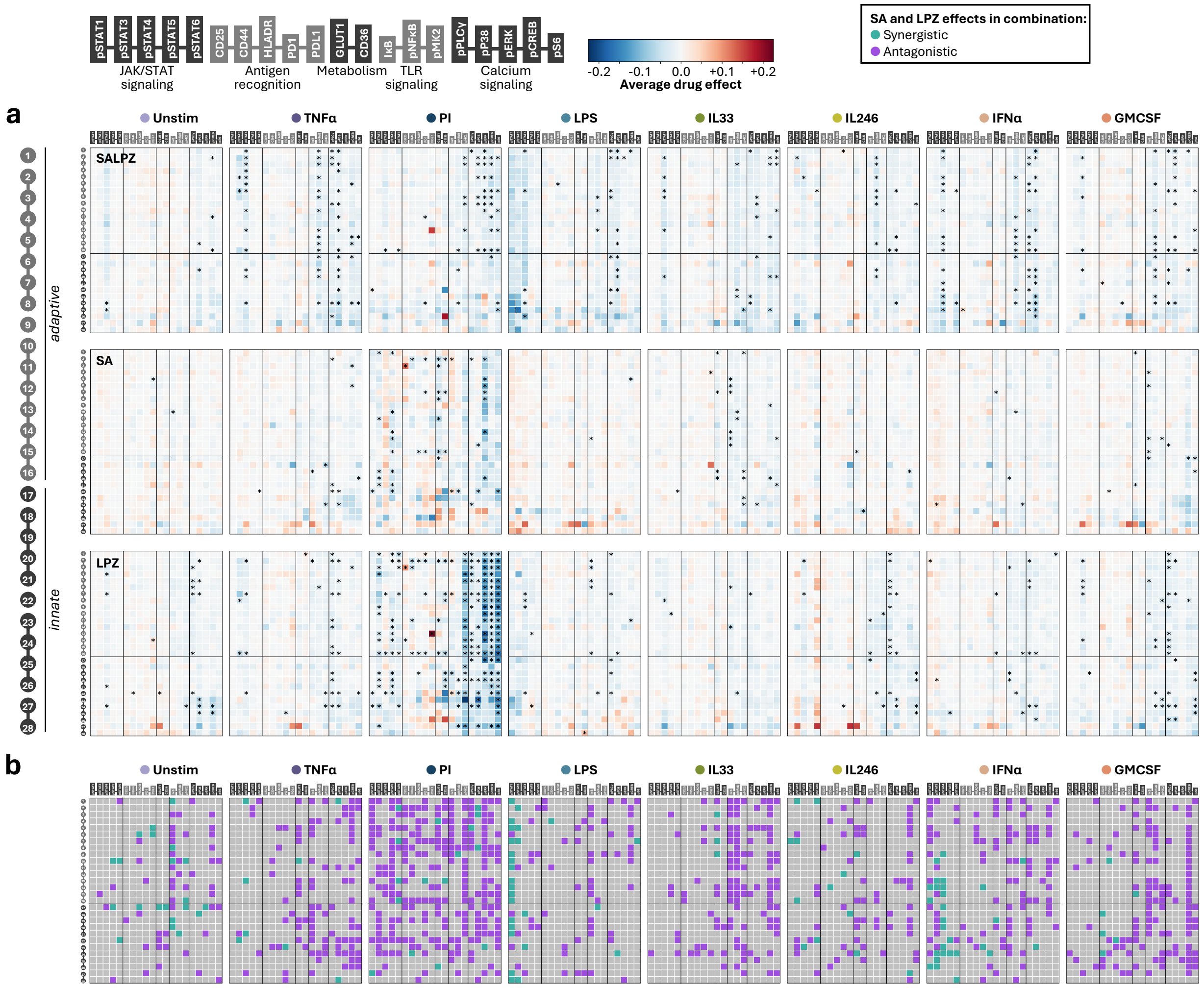

Figure S5 | Synergistic, additive, and antagonistic effects of SA, lansoprazole, and SALPZ.

a. Average drug effects mediated by SALPZ (top), SA (middle), and lansoprazole (LPZ, bottom) on n = 11 pregnant individuals.

b. Comparing the observed effects of SALPZ to the summed effects of SA and lansoprazole classified the effects as synergistic, additive, or antagonistic. Across all features, SALPZ effects were synergistic in 3%, additive in 79%, and antagonistic in 18%.

a

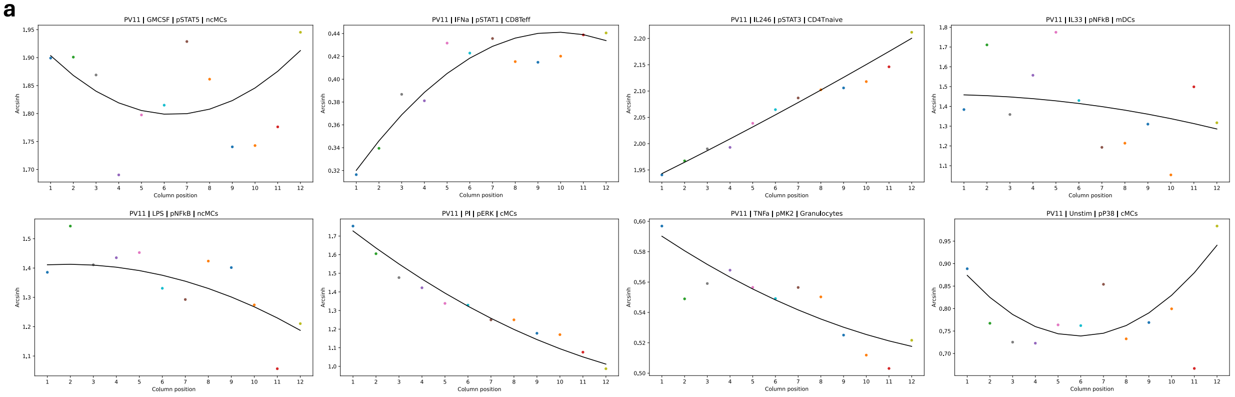

b

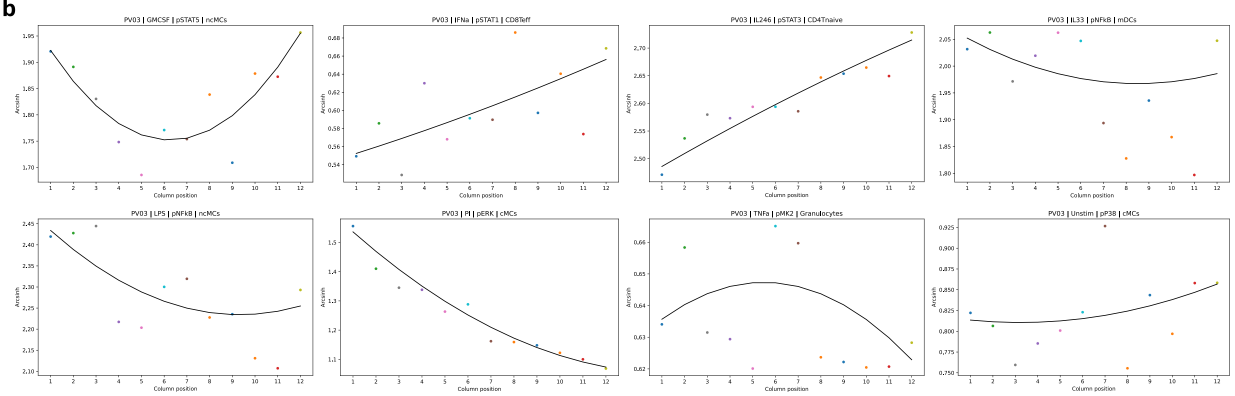

Figure S6 | Modeling of time-dependent effects on signaling response across columns. For individuals PV11 (**a**) and PV03 (**b**), the modeled time-dependent effects on signaling responses across columns is shown for eight example features. Per individual and per feature, a smooth spline was fitted across the feature values of all columns (k = 2, smoothing = 1.5, weight = 1 for drug-columns, weight = 2 for DMSO columns) to account for time-dependent effects in signaling response. Drug treatments were organized as depicted in Figure 2a. DMSO columns were placed in column 1, 6, and 12.

Target	Clone	Supplier	Catalog #
CD235ab	HIR2	Biolegend	306615
CD61	VI-PL2	BD	555752
CD45	HI30	Biolegend	304045
CD66	CD66a-B1.1	BD	551354
CD7	M-T701	BD	555359
CD19	HIB19	Biolegend	302247
CD45RA	HI100	Biolegend	304143
CD11b	ICRF44	Biolegend	301337
CD4	RPA-T4	Biolegend	300541
CD8a	RPA-T8	BD	557084
CD11c	Bu15	Biolegend	337221
CD123	6H6	Biolegend	306027
PD-1	D4W2J	CST	63815
CD44	IM-7	Biolegend	103065
CD36	D8L9T	CST	39914
PD-L1	E1L3N	CST	85164
GLUT1	EPR3915	Abcam	ab252403
CD16	3G8	Biolegend	302051
CD25	M-A251	Biolegend	356102
CD3	UCHT1	Biolegend	300443
CD62L	DREG.200	Thermo Fisher	BMS1015
CCR2	K036C2	Biolegend	357202
HLA-DR	L243	Biolegend	307651
CD14	M5E2	Biolegend	301843
CD56	NCAM16.2	BD	559043
pCREB	87G3	CST	9198
pSTAT5	C11C5	CST	51879SF
pp38	36/p38	BD	612281
pSTAT1	14/P-STAT1	BD	612132
pSTAT3	M9C6	CST	74309SF
pS6	D57.2.2E	CST	4858
pMAPKAPK2	27B7	CST	3007
Tbet	4B10	Thermo Fisher	14-5825-82
cPARP	F21-852	BD	552597
FoxP3	PCH101	Thermo Fisher	14-4776-82
IκB	L35A5	CST	4814
pNFκB	K10-895.12.50	BD	558393
pERK1/2	D13.14.4E	CST	45899SF
pSTAT6	A15137E	Biolegend	686002
pPLCγ2	A17025A	Biolegend	612402
pSTAT4	38/P-STAT4	BD	612739

Table S1 | Antibody panel of the perturbation atlas mass cytometry experiments.

Stimulation	Average MAE	Average MMD	Average Spearman R
GMCSF	0.07732349	0.01001739	0.81835463
IFNα	0.08200975	0.01234101	0.78866706
IL246	0.08949932	0.01130042	0.8079325
IL33	0.03352717	0.00247631	0.93522383
LPS	0.1246932	0.02581156	0.57759379
PI	0.14132662	0.02632193	0.72555276
TNFα	0.04773449	0.00462354	0.90259611

Table S2 | CellOT performance in cross-validation across stimulation conditions.

Model	AUROC	Pearson R	R2	Global RMSE	RMSE term	RMSE preterm
Simulated stim features (SIMCO) + Unstim features (Stelzer)	0.729	0.64	0.42	24.12	23.98	25.4
Simulated stim features (SIMCO) + Unstim features (Stelzer) + EGA	0.857	0.95	0.9	10.16	8.27	20.7
EGA	0.5	0.94	0.83	12.83	6.85	35.68
Stim features (Stelzer) + Unstim features (Stelzer)	0.788	0.75	0.55	21.14	21.57	16.4
Unstim features (Stelzer)	0.695	0.4	0.15	29.16	29.48	25.9
Simulated stim features (SIMCO)	0.715	0.64	0.4	24.38	24.2	25.95
Simulated stim features (SIMCO) + Stim features (Stelzer) + Unstim features (Stelzer)	0.805	0.8	0.62	19.41	19.68	16.5
Simulated stim features (SIMCO) + Stim features (Stelzer) + Unstim features (Stelzer) + EGA	0.9	0.95	0.9	9.96	8.72	17.8

Table S3 | Predictive performances for simulated data by SIMCO, original data by Stelzer et al. 2021, and EGA.

**Stabl grid search**	
**Hyperparameters**	**Values**
Base estimator	[Lasso, Alasso, EN]
Final model fitted	[Linear regression, Random Forest, XGBoost]
Artificial noise	[Knockoff, Random permutation]
Noise proportion	[0.8, 1.0]
**XGBoost grid search (final layer)**	
**Hyperparameters**	**Values**
n_estimators	[300, 500]
max_depth	[2, 4, 10]
learning_rate	[0.01, 0.05]
subsample	[0.5, 0.7]
colsample_bytree	[0.5,0.8]
gamma	[0, 1]
reg_alpha	[0]
reg_lambda	[1]

Table S4 | List of hyperparameter grid search for Stabl/XGBoost models.
